# Fine-Grained Ship Recognition from the Horizontal View Based on Domain Adaptation

**DOI:** 10.3390/s22093243

**Published:** 2022-04-23

**Authors:** Shicheng Sun, Yu Gu, Mengjun Ren

**Affiliations:** School of Automation, Hangzhou Dianzi University, Hangzhou 310018, China; 201060067@hdu.edu.cn (S.S.); 202060177@hdu.edu.cn (M.R.)

**Keywords:** fine-grained ship recognition, domain adaptation, computer simulation, vision transformer, local maximum mean discrepancy

## Abstract

Ship recognition is a fundamental and essential step in maritime activities, and it can be widely used in maritime rescue, vessel management, and other applications. However, most studies conducted in this area use synthetic aperture radar (SAR) images and space-borne optical images, and those studies utilizing visible images are limited to the coarse-grained level. In this study, we constructed a fine-grained ship dataset with real images and simulation images that consisted of five categories of ships. To solve the problem of low accuracy in fine-grained ship classification with different angles in visible images, a network based on domain adaptation and a transformer was proposed. Concretely, style transfer was first used to reduce the gap between the simulation images and real images. Then, with the goal of utilizing the simulation images to execute classification tasks on the real images, a domain adaptation network based on local maximum mean discrepancy (LMMD) was used to align the different domain distributions. Furthermore, considering the innate attention mechanism of the transformer, a vision transformer (ViT) was chosen as the feature extraction module to extract the fine-grained features, and a fully connected layer was used as the classifier. Finally, the experimental results showed that our network had good performance on the fine-grained ship dataset with an overall accuracy rate of 96.0%, and the mean average precision (mAP) of detecting first and then classifying with our network was 87.5%, which also verified the feasibility of using images generated by computer simulation technology for auxiliary training.

## 1. Introduction

The rapid and accurate recognition of ship targets on the surface of the sea is of great practical significance in assisting ship rescue, fishery management, sea area situation awareness, and territorial defense [1]. Knowledge guidance, template matching, and supervised classification, which rely on the artificial extraction of image features to a great extent, are usually used to detect ship targets in traditional ship recognition methods [2]. The performance of these algorithms can decrease due to illumination changes, clouds, and other factors. In recent years, with the development of big data and deep learning technologies, convolutional neural networks (CNN) have been widely used in the field of computer vision, and technology for the detection of ship targets on the sea has also seen rapid development and breakthroughs.

At present, commonly used sensors for ship detection on the surface of the sea include SAR [3,4,5,6], infrared [7,8], and visible sensors [8,9,10,11,12]. The SAR sensor has good penetrability and can work day and night; however, the ship target in SAR images is susceptible to sea clutter. In addition, ship target detection can also be affected by icebergs [6], since they look quite similar in SAR images. According to the thermal radiation characteristics of the object, the infrared sensor has the advantage of good target indication but the drawbacks of low resolution and a lack of texture. Visible sensors are easily affected by weather, and the appearance of the ship varies greatly as its angle changes when viewed from the horizontal direction. However, due to its production of high-resolution visible images containing rich spectral information and semantic details, the visible sensor is now a necessary means of reconnaissance and surveillance tasks.

For ship classification on the sea surface, warcrafts are one type of ship that is often considered. Yao et al. [9] established a ship classification collection with high-resolution optical remote sensing images that consisted of 23 categories of ships, including air carriers, destroyers, cruisers, etc. Liu et al. [10] attempted to divide ships into four categories: warcrafts, aircraft carriers, merchant ships, and submarines. Furthermore, these categories have a wealth of types, such as Arleigh Burke and Ticonderoga for warcrafts and Tarawa and Nimitz for aircraft carriers. With the deepening of the division, the category labels gradually convert from a coarse-grained level to a fine-grained level. Fine-grained classification pays more attention to the distinction of each subclass of object in the parent class. The images in the fine-grained dataset usually have the characteristics of small differences between classes and large differences within classes. For example, for the detection and recognition of ship targets at the type level, the main problem is that the results are influenced greatly by the angle of the ship during detection. There are large differences between the ship images of the same class at different angles, while the differences between the ship images of different classes may be small.

At present, there is little work on fine-grained ship recognition viewed from the horizontal direction. Because multi-angle situations increase the difficulty of fine-grained ship classification, a great deal of ship target image data with different horizontal angles must be obtained before training. However, it is usually difficult to obtain sufficient labeled data, resulting in a lack of available relevant datasets, which affects training results. In view of the above problems, this paper proposes a multi-angle ship recognition model based on computer simulation and domain adaptation. The main contributions of this paper are as follows:

- In view of the small number of subclass images of each ship type, in order to enrich the data, we propose the use of computer simulation software to generate simulation ship images with different types and different angles, so as to expand training data. The main types identified in this paper are Arleigh Burke, Murasame, Nimitz, Ticonderoga, and Wasp. Some of them were selected from the third level of [10]. Because Murasame is similar to Arleigh Burke, it was also added to verify the effectiveness of our model.
- Due to the large gap between the simulation images and the real images, we propose that style transfer be performed on the simulation images first, such that the images processed by style transfer are closer to the real ship images.
- In view of the different feature distribution between the simulation images and the real images, we propose a domain adaptation method for transfer learning. The simulation images are used as the source domain data and the real images are used as the target domain data for training. LMMD is used to realize the alignment of sub-domains and enhance the capture of fine-grained information of each category by the network, improving the recognition accuracy of the model in fine-grained classification tasks. At the same time, the feature extraction module of the transformer structure is used to extract features, and its effectiveness in fine-grained classification is verified.

## 2. Related Work

### 2.1. Fine-Grained Visual Categorization (FGVC)

For fine-grained object detection, there are two methods which are usually employed: (1) direct detection to obtain the locations and sub-category labels of the targets or (2) first, locate all targets belonging to the parent category by detection and then identify those targets to further obtain specific subclass categories. The former is better than the latter in training and inference speed; however, it is difficult to obtain satisfactory accuracy in fine-grained problems. Therefore, in the face of the problem of fine-grained image detection with high precision requirements, the method of first using detection and then further identification is a better choice. For fine-grained image classification and recognition, the following four methods can be used: (1) local features-based methods [13,14]; (2) feature encoding-based methods [15,16]; (3) attention mechanism-based methods [17,18]; and (4) transfer learning-based methods [19,20,21]. Methods based on the local features of objects focus on training a detection network to locate the local area with more discrimination capability and usually need to label the local features of the target in image; examples of these methods include part-based R-CNN [13], proposed by Zhang et al., and deep localization, alignment, and classification (LAC) [14], proposed by Lin et al. However, such methods need to consume a great deal of human resources for image annotation, and the selection of local areas also requires relevant expert knowledge as an auxiliary. Methods based on feature coding increase the dimension of image features to obtain high-order features. Bilinear CNN [15], proposed by Lin et al., uses the second moment information to externalize the output features of the two networks through bilinear pooling, thus obtaining a stronger feature representation than the linear model. However, the use of feature coding is can easily cause dimension explosion, and there will be a large number of parameters to be optimized in the model; thus, compact bilinear pooling [16] and other methods have been proposed to optimize this problem. Methods based on attention mechanisms rely on the network itself to calculate the differentiated regions to extract fine-grained information. Compared with methods based on local features, this method no longer requires component labeling and can be trained only by category labels. For example, discriminative filter learning (DFL)-CNN [17] designs a CNN framework that does not require additional parts or border annotations to learn discrimination in an end-to-end manner, allowing the network to focus exclusively on classification. Based on ViT, TransFG [18] is a transformer architecture model for processing fine-grained identification tasks. The overlap operation is used to segment the input image to reduce the loss of fine-grained information, and contrastive loss is introduced to reduce the similarity of different categories, such that the network can learn fine-grained features better. Compared with methods that require considerable resources, methods based on transfer learning can apply the knowledge learned in similar fields to the target domain, avoid a great deal of human resources invested in the annotation of new datasets, and improve the robustness and generalization of the trained recognition model. Domain adaptation is a special case of transfer learning which assumes that the source domain is different from the target domain and the source task is the same as the target task; the labeled data in the source domain is used to complete the execution of the target task. In recent years, deep domain adaptation models, such as deep adaptation networks (DANs) [20] and joint adaptation networks (JANs) [21], have been proposed to solve transfer learning problems in related fields.

### 2.2. Detection and FGVC on Ships

Growing attention has been placed on the detection of ships, and numerous methods have been proposed to solve these tasks. The existing techniques are primarily categorized into traditional handcrafted feature-based and deep learning-based methods. The traditional handcrafted feature-based methods process is usually divided into five stages: image preprocessing, region selection, handcrafted feature creation, feature extraction, and target classification. There are many works based on local handcrafted features, including local binary patterns (LBPs) [22], Hu moments with linear discriminant analysis (LDA) [23], and scale-invariant feature transform (SIFT) [24]. However, these traditional methods rely too much on handcrafted features, and the selection of the correct parameters and features requires numerous human resources and relevant expertise. Recently, deep learning-based methods have provided impressive results in multiple fields of computer vision. Nie et al. [25] added a bottom-up structure to the FPN structure of Mask R-CNN and used the spatial attention mechanism to allow the feature maps to better respond to the target’s features. Zhao et al. [26] designed a two-stage neural network that conducts ship detection as a classification problem by detecting and identifying key parts of a ship. Zhang et al. [27] proposed an attribute-guided multilevel enhanced feature representation network (AMEFRN) to fuse reweighted regional features in order to focus more on the silent region and suppress the other regions.

Although there are some studies on fine-grained ship detection and classification [8,9,10,11,27], most of this work is based on optical remote sensing images [9,10,27], and few studies have been completed on the visible classification of ships on the sea surface when viewed from the horizontal direction. Horizontal perspective ship classification is greatly affected by the angle of the ship, and many features cannot be extracted from images at certain angles. In addition, the fine-grained classification tasks should be performed based on the data; however, the relevant fine-grained datasets are lacking. In [10], an HRSC (high resolution ship collection) dataset was built, where a three-level hierarchy for ship recognition was defined, including ship class (level 1), ship category (level 2), and ship type (level 3). Warships was one of the important categories of ship, and its specific types were divided into level 3, including Arleigh Burke, Ticonderoga, and Nimitz, which were also chosen in this study. Compared with level 1 and level 2, the mAP value of level 3 detection tasks was still relatively low, indicating that the fine-grained task was still a major difficulty.

## 3. Construction of Fine-Grained Ship Dataset

Real images are essential for a fine-grained dataset, yet at the same time they are associated with certain problems. On the one hand, if the number of real images is insufficient, it is difficult to meet the needs of fine-grained problems; on the other hand, it is usually difficult to obtain real ship images at any azimuth angle. Simulation images play a crucial role when faced with the above problems. The computer simulation software can set the angle of the 3D ship model and generate a large number of simulated images to augment the dataset. Therefore, in addition to real images, our ship dataset also consisted of simulation images that were generated using computer simulation technology (shown in Section 3.2). We selected five ship categories, including Arleigh Burke, Murasame, Nimitz, Ticonderoga, and Wasp. Due to the extensive existence of these five categories of ships, it was possible to construct a fine-grained ship that could be used for detection and classification.

### 3.1. Annotation from Real Images and Videos

**Figure 1:**
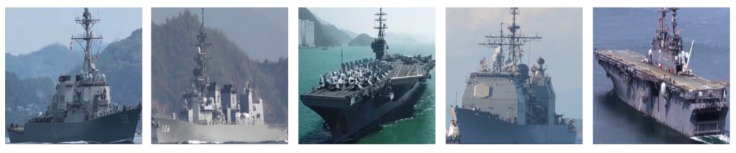
Real ship images.

In order to construct and augment the dataset, it is very important and essential to collect and annotate real ship images first. The methods of collecting real ship images mainly used in this study are as follows: (1) Five ship classes were retrieved through the search engine, and 150 images were returned from each class. The retrieved images were screened, and the images that did not conform to and/or repeat were omitted in order to obtain 100 real ship images from each class. However, even if the number of returned images continued to increase, most subsequent images were inconsistent or duplicates; thus the number of real images obtained by this method was limited. (2) On the basis of the former method, in order to continue to expand the number of real ship images in the dataset, we obtained a certain amount of five classes of ship videos through search engines, and extracted key frames containing ship targets from the videos using a semi-automatic video target annotation algorithm [28], where the target of interest is selected manually in first frame, detection and tracking algorithms are combined to locate its pixel coordinates in successive frames, and the annotation process is stopped automatically according to the output of the tracking algorithm. In order to make the difference between the extracted key frame images relatively large, multiple appropriate frame segments containing ship targets were selected in the video, and 10 key frames were extracted from each frame segment with an average selection strategy to obtain the real ship image. Thus, the number of real ship images was greatly expanded by this method. For the later object classification task, the ship targets were cropped from the images according to the annotation information. The real images obtained are shown in Figure 1.

### 3.2. Generation through Computer Simulation Technology

The number of unlabeled ship images obtained by collecting ship videos or images is still limited, and the annotation work is time consuming and laborious. Therefore, computer simulation technology was also used to generate ship images to enrich the ship dataset.

**Figure 2:**
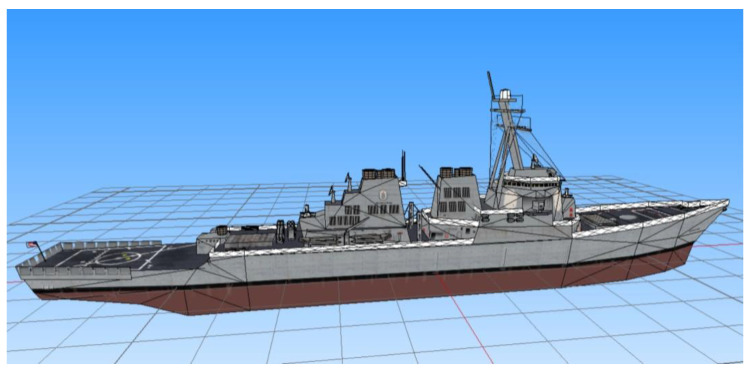
3D modeling of ship target.

**Figure 3:**
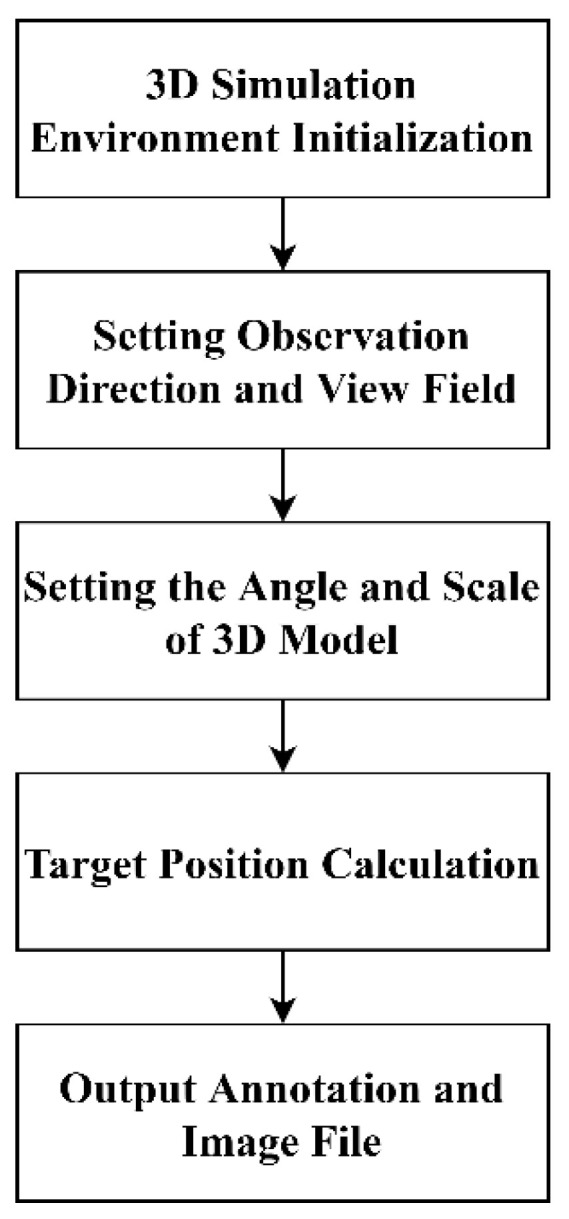
Ship image generation process based on computer simulation.

The 3D model of one class of ship is shown in Figure 2. When using computer simulation technology to render a 3D scene, the specific process can be described as follows, and is shown in Figure 3: (1) First, a 3D model of the ship target is loaded after initializing a 3D scene when establishing the marine simulation environment. (2) Second, the direction and angle of the view is set. (3) Third, the rotation angle of the ship 3D model object around the Z axis and the scaling ratio of the target is set. (4) Fourth, the bounding box of the target is calculated based on collision detection theory, and the pixel coordinates of the target are obtained. The category information is obtained via the 3D model. (5) Finally, the simulation image and the corresponding annotation file are written to the disk.

**Figure 4:**
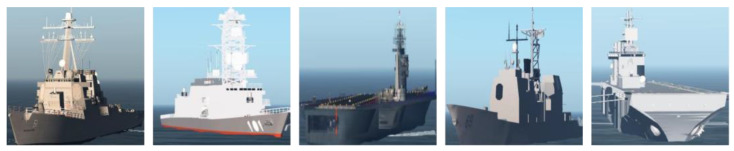
Simulation ship images.

The computer simulation software can set various environmental factors, including time, visibility, and weather, which control the pixel intensities of the whole image during the 3D simulation environment initialization; it can also set the position, direction, and scale of the simulation target to obtain ship simulation images and annotation files of different scenes, angles, and sizes. We also cropped the ship targets from the simulation images according to the annotation information for classification. The generated simulation images are shown in Figure 4.

### 3.3. Statistical Analysis of Ship Target Datasets

When constructing the ship dataset, in addition to environmental factors, such as weather and illumination, the azimuth angle of the ship is also an important factor affecting the detection performance. Although the distribution of ship samples can be enriched by technologies, such as key frame extraction, collecting images of multiple categories of ships with different angles is still difficult. Therefore, the visibility value, time point (illumination), and target angle are three main parameters need to be set in the simulation process.

The specific dataset information is shown in Table 1. We combined two time points and three visibility values to obtain six different combinations; for each combination, one image was captured for each rotation degree of the 3D model, i.e., 360 images were obtained after one rotation. Thus, a total of 2160 simulation images were obtained for each class. Then, some samples were selected and extracted from the real samples to form the test set. All datasets were composed of five common warships in different scenes or from different angles; the number of images in each class was the same on average to ensure class balance. Because the images in the simulation dataset were generated by the simulation software according to the 3D model, there was a large difference in the marginal probability distribution between them and the real images.

**Figure 5:**
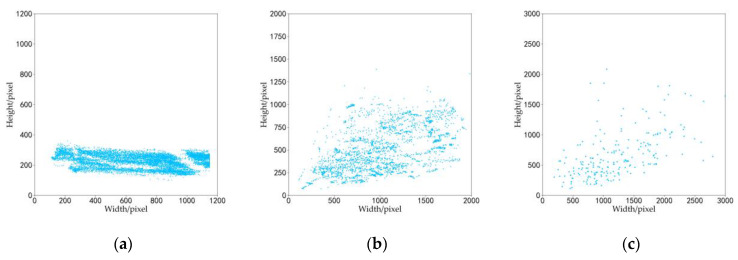
Target size scatter plot for the (**a**) simulation dataset, (**b**) real dataset, and (**c**) test dataset.

The target size scatter plots for the three datasets are shown in Figure 5. The target size of the simulation dataset was concentrated and evenly distributed between 150 × 200 and 1150 × 300 pixels, and the target size of the real dataset was mainly concentrated within 1100 × 600 pixels. Since the dataset images are a variety of ship targets, when the horizontal perspective is biased towards the head or tail of the ship, the height of the target size may be greater than the width, and the width of the target size is usually greater than the height in other cases. Especially for Nimitz, the ratio of width to height was larger.

## 4. Ship Classification Based on Domain Adaptation and Vision Transformer

We can train a detection model based on a self-established dataset, which can be used directly for fine-grained ship detection, or we can train a recognition model to further classify the objects detected by other detectors. Considering the characteristics of fine-grained vision tasks, it is difficult to obtain desirable results through direct end-to-end detection; thus, we chose the latter method in this study.

**Figure 6:**
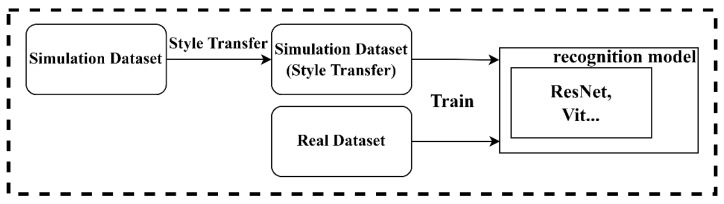
Training process.

Since there was a large gap between simulation dataset and real dataset, the preprocessing style transfer operation was performed first, then the output simulation dataset was used to assist the real dataset for recognition model training. The recognition model training process is shown in Figure 6.

For the current two sources, there are two strategies for training. One is the mix strategy, which directly mixes the two datasets for training. The other is the transfer learning strategy, which transfers the knowledge in the source domain to help the target domain with training. Due to the distribution difference between the two datasets, it was not appropriate to directly mix the simulation images and real images for training. If the features extracted by the feature extractor are biased towards a certain domain, the classification results in other domains will be affected; on the contrary, it is beneficial for classification when the extracted features have common characteristics in different domains. Therefore, domain adaptation architecture was adopted to train the ship recognition model. The simulation images (source domain) and real images (target domain) were used for training to obtain a feature extractor and classifier that could accurately classify real test data, and the maximum mean discrepancy (MMD) was used to measure and reduce the difference between simulation data features and real data features after they were mapped to high-dimensional space, such that the feature extractor could extract domain-invariant features.

### 4.1. The Architecture of Training Ship Recognition Model Based on Domain Adaptation

Here, the definition of domain adaptation and related symbols will be introduced based on [29]. When given a source domain $D_{s}$ and a source task $T_{s}$, as well as a target domain $D_{t}$ and a target task $T_{t}$, transfer learning is used to help improve the optimization of the target prediction function f(·) in $D_{t}$ by using the knowledge in $D_{s}$ and $T_{s}$, where $D_{s}\neq D_{t}$ and $T_{s}\neq T_{t}$.

Domain adaptation is a special case of transfer learning, known as transductive transfer learning. It assumes that the source domain is different from the target domain, and the source task is the same as the target task, i.e., $D_{s}\neq D_{t}$ and $T_{s\;}=T_{t}$. Domain adaptation usually uses labeled data from a single or multiple related source domains to complete the execution of the target task. When there are domains with different but similar distributions, the domain adaptation method can transfer the knowledge learned in them, avoiding the use of considerable human resources for the annotation of datasets and improving the robustness and generalization of the trained model.

**Figure 7:**
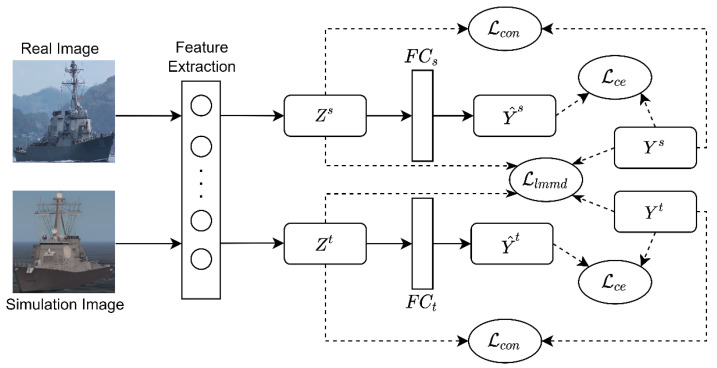
The architecture of training recognition model based on domain adaptation.

In this study, a recognition model based on a deep sub-domain adaptive network [19] was used. On this basis, the characteristics of the ship fine-grained dataset and the transformer model [23] suitable for fine-grained recognition were comprehensively considered, and the training process and recognition model architecture were designed. The specific architecture is shown in Figure 7.

### 4.2. Ship Recognition Model

Transformers [30] have made outstanding progress in natural language processing (NLP) and machine translation. Inspired by this, researchers have attempted to apply transformers to computer vision tasks, such as image classification [31], object detection [32] and semantic segmentation [33]. A transformer has a weaker inductive bias than a CNN, and its characteristics enable it to use global information at the beginning of the process; thus, it usually requires larger datasets to achieve better training results. 

**Figure 8:**
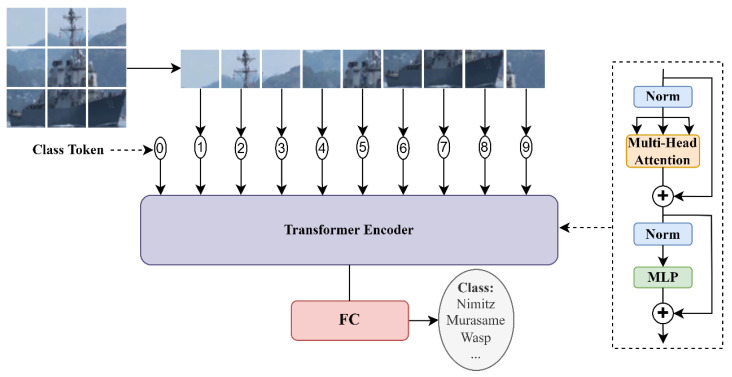
ViT model overview.

ViT [31] is the first model to directly apply the native transformer structure on NLP to image classification. In order to match the input of the transformer, as shown in Figure 8, the input image $x\in {\mathbb{R}}^{H\times W\times C}$ is divided and flattened into a sequence of 2D patches $x_{p}\in {\mathbb{R}}^{{N\times (P}^{2}\cdot C)}$, where the resolution of each patch is P×P and the number of patches is $N={HW/P}^{2}$. Then, the patches are mapped to D dimension to obtain patch embeddings $x_{p}^{*}\in {\mathbb{R}}^{N\times D}$. ViT adds position embeddings on the basis of patch embeddings to retain position information, and an additional vector (class token) is also embedded for subsequent classification tasks. The transformer encoder block consists of multi-head self-attention (MSA) and MLP stacked, and the features corresponding to the class token are extracted by these blocks for classification. Since the transformer has a better transfer effect than a CNN, ViT can be transferred to small datasets after pretraining and can achieve excellent classification performance. As a pure model of visual tasks using a transformer, ViT has also become the starting point for many subsequent works [23,34].

In this study, the source domain $D_{s}={\{(x}_{i}^{s}{,y}_{i}^{s})\}$ consisted of n labeled simulation ship images which were processed by style transfer, while the target domain $D_{t}={\{(x}_{i}^{t}{,y}_{i}^{t}{)}_{i=1}^{m}\}$ consisted of m labeled real ship images. Feature ${z=f}_{e}\left({x;\theta }_{e}\right)$ was extracted by feature extraction network $f_{e}$, and then predicted label ${\hat{y}=f}_{c}\left({z;\theta }_{c}\right)$ was obtained by classifier $f_{c}$, where $\theta_{e}$ and $\theta_{c}$ are parameters of the feature extraction network and classifier, respectively.

### 4.3. Loss Function

The loss function in the model is mainly composed of the following three parts. Firstly, the cross-entropy loss function is applied to calculate the classification loss in source domain and target domain as follows:

(1) $$L_{ce}=\frac{1}{n}\sum_{i=1}^{n}J\left({\hat{y}}_{i}^{s},y_{i}^{s}\right)+\frac{1}{m}\sum_{i=1}^{m}J\left({\hat{y}}_{i}^{t},y_{i}^{t}\right)$$

where J(,) is the cross-entropy loss function.

Since the difference between sub-categories in fine-grained data is very small, it is not enough to rely solely on cross-entropy loss to supervise the learning of features. Therefore, additional contrastive loss [23] should be added to minimize the similarity of different classes and maximize the similarity in the same class. The loss function is as follows:

(2) $$\begin{array}{cc} L_{con}= & \frac{1}{b_{s}{}^{2}}\sum_{i=1}^{b_{s}}\left[\sum_{j:y_{i}^{s}=y_{j}^{s}}^{b_{s}}\left(1-Sim\left(z_{i}^{s},z_{j}^{s}\right)+\sum_{j:y_{i}^{s}\neq y_{j}^{s}}^{b_{s}}max\left(Sim\left(z_{i}^{s},z_{j}^{s}\right)-\alpha ,0\right)\right)\right]\\ & +\frac{1}{b_{t}{}^{2}}\sum_{i=1}^{b_{t}}\left[\sum_{j:y_{i}^{t}=y_{j}^{t}}^{b_{t}}\left(1-Sim\left(z_{i}^{t},z_{j}^{t}\right)+\sum_{j:y_{i}^{t}\neq y_{j}^{t}}^{b_{t}}max\left(Sim\left(z_{i}^{t},z_{j}^{t}\right)-\alpha ,0\right)\right)\right] \end{array}$$

where $b_{s}$ and $b_{t}$ are the batch size of the source domain dataset and target domain dataset, respectively; $z_{i}$ and $z_{j}$ are normalized by $l_{2}$; and ${Sim(z}_{i}{,z}_{j})$ is the dot product of $z_{i}$ and $z_{j}$. *α* is a constant, and only negative pairs with ${Sim(z}_{i}{,z}_{j})$ larger than *α* will contribute to the loss $L_{con}$.

When using the discrepancy-based method to align the statistical distribution of the source domain and target domain and reduce the distribution difference, MMD is a common practice. Ghifary et al., proposed an MMD metric model for feedforward neural networks with a single hidden layer [35]. The empirical estimation of MMD is as follows:

(3) $$d_{H}={∥\frac{1}{n}\sum_{i=1}^{n}\phi (x_{i}^{s})-\frac{1}{m}\sum_{j=1}^{m}\phi (x_{j}^{t})∥}_{H}^{2}$$

where H is the reproducing kernel Hilbert space and ϕ(·) is the kernel function that maps the original data to the reproducing kernel Hilbert space.

Domain alignment performed without considering the relationship between subdomains of the same category in the two domains is called global alignment. However, global alignment leads to the inability of the network to capture fine-grained information, which leads to unsatisfactory results from transfer learning, especially on fine-grained datasets. Zhu et al. proposed a deep sub-domain adaptive network DSAN [19] in which local maximum mean discrepancy (LMMD) is used to align subdomains in two domains, so as to capture fine-grained information in each category. The definition of LMMD is as follows:

(4) $$d_{H}=\frac{1}{C}\sum_{c=1}^{C}{∥\frac{1}{n}\sum_{i=1}^{n}\omega_{i}^{sc}\phi (x_{i}^{s})-\frac{1}{m}\sum_{j=1}^{m}\omega_{j}^{tc}\phi (x_{j}^{t})∥}_{H}^{2}$$

where $\omega_{i}^{sc}$ and $\omega_{j}^{tc}$ are the weights of $x_{i}^{s}$ and $x_{j}^{t}$, respectively, corresponding to category c and $\omega_{i}^{c}$ is obtained by the following formula:

(5) $$\omega_{i}^{c}=\frac{y_{ic}}{\sum_{\left(x_{j},y_{j}\right)\in D}y_{jc}}$$

where $y_{ic}$ is the cth term of vector $y_{i}$.

In this study, the extracted features were mapped to reproducing kernel Hilbert space (RKHS), and the MMD between the corresponding categories was calculated. The function requires four input parameters: the output features $z^{s}$ and $z^{t}$, source domain data label $y^{s}$, and target domain data label $y^{t}$. The specific expansion is as follows:

(6) $$L_{lmmd}=\frac{1}{C}\sum_{c=1}^{C}\left[\sum_{i=1}^{n}\sum_{j=1}^{n}\omega_{i}^{sc}\omega_{j}^{sc}k\left(x_{i}^{s},x_{j}^{s}\right)+\sum_{i=1}^{m}\sum_{j=1}^{m}\omega_{i}^{tc}\omega_{j}^{tc}k\left(x_{i}^{t},x_{j}^{t}\right)-2\sum_{i=1}^{n}\sum_{j=1}^{m}\omega_{i}^{sc}\omega_{j}^{tc}k\left(x_{i}^{s},x_{j}^{t}\right)\right]$$

where ${k(x}^{s}{,x}^{t})=\langle \phi {(x}^{s}),\;\phi {(x}^{t})\rangle =\langle z^{s}{,\;z}^{t}\rangle$ and 〈,〉 is the inner product of vectors. The total loss function can be written as:

(7) $$L=L_{ce}+\lambda_{1}L_{con}+\lambda_{2}L_{lmmd}$$

where $\lambda_{1}$ and $\lambda_{2}$ are the coefficients of contrastive loss and LMMD loss, respectively.

## 5. Experiments

In this section, we first introduce the detailed setup, including the datasets and parameters, for model training. The experimental results and a quantitative analysis are then given followed by ablation experiments.

### 5.1. Experimental Setting

Due to the large distribution difference between the simulation images and real images, it can be difficult to align the source domain and the target domain during the training, which may even lead the performance of the model to degrade in the target domain. In view of this, style transfer was used in this experiment to preprocess the simulation images before training. If the gap between real images and simulation images processed by style transfer is reduced, it can prevent the occurrence of negative transfer and thereby improve the convergence speed and classification accuracy of the model.

**Figure 9:**
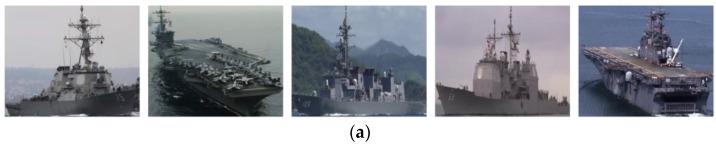

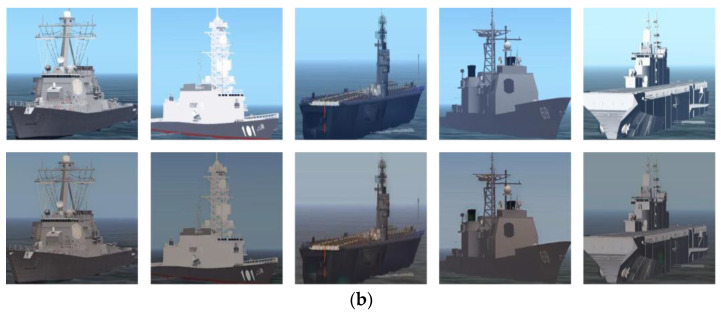
Style images and results. (**a**) Some style images selected from real samples. (**b**) Original simulation images and results processed by style transfer.

Several appropriate style images, which are shown in Figure 9a, were selected according to the real images, and the style transfer model was trained on these style images and the COCO 2017 dataset (content images). Then, we obtained the model that could transfer the style of input images to be similar to that of the style images (real ship images). Finally, the images in the simulation dataset were inferred by the style transfer model to obtain the result images. The original simulation images and output result images are shown in Figure 9b; intuitively, the simulation images processed by style transfer method are shown closer to the real images.

The backbone of recognition model was ViT-B_16 pretrained on ImageNet-21k. Then, the recognition model was trained on the simulation dataset and real dataset, and the cross-entropy loss function, LMMD loss function, and contrast loss function were used for back propagation. The training used stochastic gradient descent (SGD) as the optimizer, where the warm-up strategy is used on the learning rate setting by starting with a smaller learning rate, then switching to a larger learning rate and beginning to decay. This helps to keep the distribution stable, avoid overfitting in advance, make the learning rate adapt to different sizes of training sets, and accelerate the convergence of the model. The specific parameter settings are shown in Table 2.

In order to further increase the diversity of the training samples and improve the robustness and generalization ability of the model, in addition to style transfer, we made other data augmentations for training samples by random scaling, clipping, horizontal flip, brightness, saturation, and contrast changes.

### 5.2. Experimental Results

#### 5.2.1. Ablation Experiments

This section analyzes and evaluates the accuracy performance of the model obtained by the training process in this paper on the ship fine-grained dataset, as well as the results of related ablation experiments. The experimental results are shown in Table 3, and the accuracy of each class is shown in Table 4. In the Table 3, overall accuracy (OA) is the ratio of the number of samples correctly classified in the test set to the number of samples in the test set. The specific mathematical formulas of the metrics used are as follows:

(8) $$OA=\frac{\sum_{i=1}^{C}TP_{i}}{\sum_{i=1}^{C}N_{i}},\;Acc_{i}=\frac{TP_{i}}{N_{i}},\;AA=\frac{\sum_{i=1}^{C}Acc_{i}}{C}$$

where C is the number of categories, TP is an outcome where the model correctly predicts the positive class, and N is the number of samples in each category. Acc is the classification accuracy of each category. AA is the average of all Acc values.

The experimental results showed that the overall accuracy of the recognition model obtained on the ship fine-grained test set was 96.0%, which was 15.5% higher than the benchmark accuracy of 80.5% obtained by the direct recognition of the ResNet50 model. It can be seen from Table 3 that the accuracy of the recognition model obtained by directly mixing simulation images and real images was lower than the baseline. Although the amount of data increases after mixing, the style difference between the simulation images and the real images is too large, resulting in a decline in the performance of the trained model on the test set. After adding style transfer, the accuracy rate rebounded, precisely because the style deviation between the simulation images and the real images was reduced.

After using global alignment and sub-domain alignment, the recognition accuracy of the model was improved. Overall accuracy rates of 90.7% and 92.5% were achieved without style transfer in the simulation dataset, indicating the effectiveness of the images obtained by computer simulation software for assisting the real images in fine-grained recognition. After adding the style transfer, the recognition accuracy of the two increased by 1.7% and 0.9%, respectively, implying that the use of style transfer reduces the difference between the simulation images and the real images, which is more conducive to the subsequent transfer learning. Compared with the recognition model obtained by global alignment, the recognition model obtained by sub-domain alignment was improved by 1.8% and 1.0% before and after style transfer, respectively. Because sub-domain alignment can capture more fine-grained information, it has better performance on ship fine-grained datasets.

The self-attention mechanism of the transformer weights and aggregates individual patches of the input image, enabling it to capture important regions in the image. Compared with a CNN, a transformer can obtain remote global information in the shallow layer, which is a natural advantage for dealing with fine-grained classification problems. After the ResNet50 module in each model was changed to the ViT-B_16 transformer structure in the experiment, the accuracy of the recognition model was improved. In the case of only using a real image dataset, the result of ViT-B_16 was 85.4%, which was about 6% higher than the reference value of ResNet50, indicating the effectiveness of the transformer on fine-grained datasets. The overall recognition accuracy after domain adaptation training combined with the simulation images after style transfer for subdomain alignment was as high as 96%, which was 2.6% higher than that obtained using ResNet50 as the backbone network.

**Figure 10:**
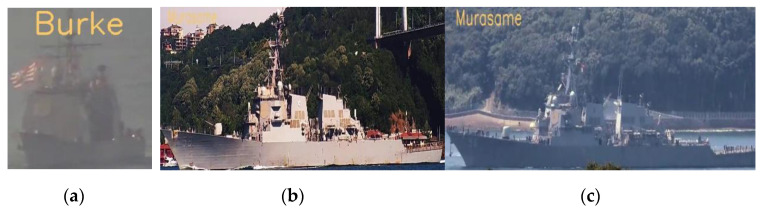
Misclassification examples: (**a**) misclassifying Ticonderoga as Arleigh Burke; (**b**) misclassifying Arleigh Burke as Murasame; (**c**) misclassifying Arleigh Burke as Murasame.

Figure 10 shows some examples of misclassification. When the perspective is biased towards the head or tail of the ship, the difference between similar ships will become very small, and the classifier will have more misclassifications at this time. For example, the classifier misclassified Ticonderoga in Figure 10a as Arleigh Burke. When the background is complex, it will also affect the recognition results to a certain extent. As shown in Figure 10b,c, the mast part of the ship was affected by the complex background, and the classifier misclassified Arleigh Burke as Murasame.

#### 5.2.2. Detection and Classification Results

Deep object detection models can be divided into one-stage and two-stage models according to the number of stages in the detection network. Among them, one-stage representatives include the YOLO (you only look once) series [36,37], single shot detection (SSD) [38], etc., and two-stage representatives include a series of detection models represented by Faster RCNN [39]. Usually, one-stage object detection models have a faster inference speed and can meet the real-time requirements. According to whether Anchor is used in the model, it can be divided into two categories: Anchor-Based and Anchor-Free. Common Anchor-Free models include CornerNet [40], fully convolutional one-stage object detector (FCOS) [41], etc., which effectively alleviate the problems caused by using Anchor, such as the need to set too many hyperparameters and the imbalance of positive and negative samples. In addition, transformer-based detection models, such as DETR [32] and Deformable DETR [42], are also being developed. Among them, YOLOv5 has superior performance, fast reasoning speed, and strong flexibility, and is one of the best models in the field of target detection.

We further compared the performance of direct detection and first detection then classification. First, the YOLOv5s model was trained on the fine-grained dataset we established in this study for the direct detection of five fine-grained categories. We set the hyperparameters as follows: a batch size of 4, an initial learning rate of 0.001, and a training epoch of 150 times. Then, we adopted the two-stage method of first detection and then classification, in which the detector also uses the YOLOv5s model. We selected another ship dataset [28] (a total of 12,817 images consisting of warcraft samples and common-ship samples) and randomly allocated samples according to the corresponding proportion to form a training set, test set, and validation set, thus completing the construction of the detection dataset. Then, the recognition model obtained in this study was used as a classifier. The results were evaluated by mAP, which represents the mean of the area of the precision-recall (P-R) graph. It can be obtained through Equation (9), where P(r) is the function of P-R curve.

(9) $$\mathrm{mAP}=\frac{\sum_{i=1}^{C}\int_{0}^{1}P(r)dr}{C}\;$$

As shown in Table 5, the mAP was low when YOLOv5s was used directly for fine-grained detection on the test set; when first using YOLOv5s to detect warcraft and then using our method for classification to obtain fine-grained labels, the mAP was greatly improved. However, compared with the OA of the classification, the mAP was relatively low. The reason for this is that the detection results are not perfect, and many features are not included in the bounding box, which affects the classification results.

**Figure 11:**
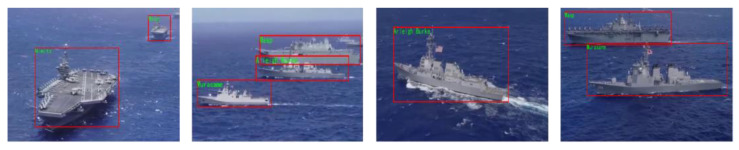
Detection and classification results.

In addition to the ship images, a video was selected for testing, and the detection and classification results are shown in Figure 11.

Due to the presence of a certain amount of small ship targets in the video, it was often difficult to classify them at a fine-grained level. In this study, the detected ship targets with an area of more than 1500 pixels were classified to obtain fine-grained labels, while for ship targets with an area of less than 1500 pixels, only the labels obtained by the detector were output.

When the ship motion in the video was relatively stable, the recognizer could obtain better results. However, when the ship rotated rapidly, the recognition result was still affected to a certain extent. Because the affine transformation occurs during the rotation of the ship, there may be a large difference between any two moments, such that the recognizer cannot obtain stable and correct results. This is one of the issues that needs to be addressed in future work.

## 6. Conclusions

This paper took the recognition and detection of maritime ships as the research background and explored the fine-grained classification of ships in visible images. In order to meet the needs of the experiment and evaluate our approach, a fine-grained ship classification dataset consisting of five categories of ships was established for this investigation. The images in this dataset, which could be divided into real images and simulation images, were collected from videos and generated by computer simulation software. A novel classification framework with domain adaptation and a vision transformer was proposed for fine-grained ship classification tasks. Essentially, the real images and the simulation images processed by style transfer were input into the network and features were extracted by ViT. Then, the features were mapped to RKHS, and the LMMD loss between the corresponding categories was calculated. The LMMD loss, contrastive loss, and CE loss were integrated to optimize the model. The experimental results indicated that simulation images could be used for training with real images, and the transformer structure showed good performance in fine-grained classification. Our network presented with high performance on the self-established fine-grained ship dataset. The results of first detection and then classification on the ship video also showed the effect of our network in specific application.

Despite the high performance of our network, the results obtained during the ship rotation process in the video were still unstable. Based on this, future research work should include modifications to the network and the exploration of other algorithms for fine-grained ship recognition.

## Figures and Tables

**Table 1:** Dataset information.

Dataset	Class	Arl.	Mur.	Nim.	Tic.	Wasp	Total
Simulation	5	2160	2160	2160	2160	2160	10,800
Real	5	545	497	519	502	505	2568
Test	5	48	45	47	43	43	226

**Table 2:** Training parameters of recognition model.

Backbone	Loss	Epoch	Optimizer	Batch Size	Learn Rate	Image Size
ViT-B_16	CE + LMMD + Contrastive	100	SGD (m = 0.9)	16	1 × 10^−2^	224 × 224

**Table 3:** Test results on ship fine-grained dataset.

ID *	Backbone	Training Strategy	Style Transfer	OA/%
1	ResNet50	Only real dataset		80.5
2	ResNet50	Directly mix datasets		76.1
3	ResNet50	Directly mix datasets	√	80.0
4	ResNet50	Domain adaptation (global alignment)		90.7
5	ResNet50	Domain adaptation (global alignment)	√	92.4
6	ResNet50	Domain adaptation (sub-domain alignment)		92.5
7	ResNet50	Domain adaptation (sub-domain alignment)	√	93.4
8	ViT-B_16	Only real dataset		85.4
9	ViT-B_16	Directly mix datasets		81.4
10	ViT-B_16	Directly mix datasets	√	84.5
11	ViT-B_16	Domain adaptation (global alignment)		93.8
12	ViT-B_16	Domain adaptation (global alignment)	√	95.6
13	ViT-B_16	Domain adaptation (sub-domain alignment)		95.2
14	ViT-B_16	Domain adaptation (sub-domain alignment)	√	**96.0**

* Table 3 and Table 4 use the same method for corresponding ID.

**Table 4:** Accuracy of each class and average accuracy.

ID *	Arl./%	Mur./%	Nim./%	Tic./%	Wasp/%	AA/%
1	89.6	82.2	61.7	69.8	**100.0**	80.7
2	62.5	73.3	68.1	81.4	97.7	76.6
3	81.3	80.0	63.8	76.7	**100.0**	80.4
4	95.8	82.2	95.7	79.1	**100.0**	90.6
5	95.8	86.7	93.6	86.0	**100.0**	92.4
6	**100.0**	84.4	91.5	93.0	95.3	92.9
7	**100.0**	86.7	**100.0**	79.1	**100.0**	93.1
8	75.0	80.0	97.9	90.7	83.7	85.5
9	77.1	60.0	95.7	83.7	90.7	81.4
10	72.9	95.6	95.7	72.1	86.1	84.5
11	83.3	93.3	**100.0**	95.3	97.7	93.9
12	89.6	93.3	97.9	97.7	**100.0**	95.7
13	81.3	95.6	**100.0**	**100.0**	**100.0**	95.4
14	85.4	**97.8**	**100.0**	97.7	**100.0**	**96.2**

* Table 3 and Table 4 use the same method for corresponding ID.

**Table 5:** Results of direct detection and first detection then classification.

Method	Arl./%	Mur./%	Nim./%	Tic./%	Wasp/%	mAP/%
YOLOv5s	81.6	81.5	61.5	58.2	78.7	72.3
Cascade R-CNN	86.5	88.6	62.4	63.0	86.1	77.3
YOLOv5s + Ours	82.1	87.6	89.6	81.4	96.9	87.5

## Data Availability

Not applicable.
